# Insulin mediated improvement in glycemic control in elderly with type 2 diabetes mellitus can improve depressive symptoms and does not seem to impair health-related quality of life

**DOI:** 10.1186/s13098-015-0052-1

**Published:** 2015-06-24

**Authors:** R. A. Oliveira, M. Tostes, V. A. Queiroz, M. Rodacki, L. Zajdenverg

**Affiliations:** Nutrology and Diabetes Section, Federal University of Rio de Janeiro, Rio de Janeiro, Brazil

**Keywords:** Health- related quality of life, Depression, Diabetes mellitus, Elderly, Insulin

## Abstract

**Background and objectives:**

Type 2 diabetes (T2D) is very prevalent among the elderly. Insulin therapy is often required for glycemic control. The association of starting this therapy with depressive symptoms as well the health-related quality of life (HRQoL) is unknown among the elderly patients.

**Aims:**

Evaluate the association of starting insulin therapy depressive symptoms as well with HRQoL of elderly people with T2D.

**Methods:**

36 T2D participants (67.9 % females, age 66.5 years ± 5.1) were recruited, 26 of whom completed the follow-up. Generic (Short-Form 36 Health Survey - SF-36) and specific (Problem Areas in Diabetes - PAID) HRQoL questionnaires, Beck Depression Inventory (BDI), clinical, laboratorial and socio-demographic data were recorded on baseline and 6 months after the beginning of insulin treatment.

**Results:**

There was a reduction in the BDI score after the use of insulin, which means an improvement in depressive symptoms (Before/After: median – 10.5 / 7; *p* = 0008). There were no statistically significant differences in HRQoL scores between the two time periods There was also a reduction in HbA1c (Before/After: median – 8.7/7.9). Otherwise, there were no statistically significant differences in: BMI (28.1/28.3); Abdominal circumference:(100.5/99.5) and chronic complications status.

**Conclusion:**

Insulin therapy in elderly people with type 2 diabetes can lead to an improvement of depressive symptoms and does not seem to affect negatively HRQoL of the participants.

## Introduction

Type 2 diabetes mellitus (T2D) is prevalent among adults, especially among elderly people. New data from International Federation of Diabetes (IDF) indicates that there are currently more than 285 million people with T2D around world. It is estimated that by 2030 this figure will have increased to more than 552 million people with T2D. Population growth and improvements in public health leading to an increase on life expectancy have contributed to a steady increase in these numbers and consequently to an increase in the number of older people with diabetes. Aged subjects with diabetes have an increased rate of diabetes-related complications and are more likely to have comorbid conditions, such as physical disability, cognitive dysfunction and depression, all of which could impair health-related-quality-of-life (HRQoL) [1].

Geriatric diabetes care guidelines recognize the importance of improving HRQoL for older subjects with diabetes. Almost all studies have compared HRQoL in older adults with T2D with HRQoL in those without T2D. Studies evaluating the impact of different types of T2D therapies on HRQoL and depressive symptoms in this population are scarce. Three central goals in the treatment of diabetes mellitus are the avoidance of hyperglycaemia (prevention of the development or progression of diabetes complications over time), the avoidance of hypoglycaemia and the maintenance or achievement of good HRQoL. Having multiple diabetic complications is clearly associated with decreased HRQoL. Data from large studies, such as the Diabetes Control and Complications Trial (DCCT) and United Kingdom Prospective Diabetes Study (UKPDS), suggest that intensive treatment itself (using insulin or not) does not impair HRQoL [2]. During past few years, interest in the associations between diabetes and HRQoL has grown considerably. In the 1970s, just 13 studies mentioned the words ‘diabetes’ and ‘quality of life’ in their abstracts. A recent research in pubmed has retrieved 8518 citations. HRQoL is defined as a multidimensional concept including not only the domains of physical health and functioning, but also concern about the future. Having diabetes for some subjects may have a different meaning which is strongly influenced by a person’s value and personality. The self-report is the best way to measure HRQol. Generic and diabetes-specific questionnaires are accepted as preferred tools for its assessment. The term HRQoL also includes physical, psychological and social domains related to the health [3].

The role of insulin therapy over HRQoL of patients with diabetes is controversial. Data from A1chieve study, which evaluated people with T2D with unsatisfactory glycemic control, has showed benefit of starting insulin on HRQoL of the sample studied. In this study, people with T2D starting insulin significantly improved overall HRQoL across all component health dimensions. However, the average age of the population in the study was 54.9 (±14.4) years. So far, we could not extrapolate these data to elderly population [4]. A systematic review and meta-analysis of 11 trials compared the effects of treatment with regular insulin versus short-acting insulin analogues over HRQoL of patients with diabetes. It concluded that objective interpretation on the results was not possible due to the various instruments used [5]. Other study, which has not been included in that meta-analysis, found that the combination insulin glargine plus insulin lispro improved HRQoL in comparison with NPH insulin and regular insulin [6]. Otherwise, in Horvath’s meta-analysis no evidence for beneficial effect of long-acting analogues on HRQoL could be observed [7].

The link between depressive symptoms and T2D remains unknown. If depression was a causal factor for the development of T2D, depressed patients would be at an increased risk of developing pre-diabetes, which is not true. A suggestion of a link between depression and diabetes comes from reports of a positive association between continuous measures of depression (eg, severity scores) and serum glucose levels (eg, fasting glucose, 2 h post-load glucose, or HbA1c concentration). However, it had also been showed that both hypoglycaemia as well hyperglycaemia were associated with higher levels of depressive symptoms, contradicting the idea of a direct link between depression and dysglycaemia [8]. The role of insulin therapy on depressive symptoms is also not well established. Although there is some evidence depressive symptoms seem to be more prevalent in subjects with T2D under insulin therapy, there also exists data showing this therapy is not associated with an increase in such risk [9, 10].

Finally, the role of insulin therapy over depressive symptoms and HRQoL in adults with T2D remains controversial. Indeed, it has not been studying specifically in elderly population so far. The aim of our study was to evaluate the role of insulin therapy on depressive symptoms as well HRQoL of elderly people with T2D. Our study is especially of value because of its prospective design.

## Subjects, materials and methods

### Patient selection and procedures

The Brazilian National Institute of Health defines as an elderly a person who is 60 years or older. Patients with 60 years or older and diabetes from the outpatient unit of nutrology and diabetes from Hospital Universitário Clementino Fraga Filho with indication of starting insulin therapy between January 2012 and June 2013 were invited to be included in the research. We adopted a convenience sample, one of the main types of non-probability sampling methods. Patients who had been included gave written informed consent to participate. Data was collected using paper and pen in the waiting room. The Research and Ethics Committee of Federal University of Rio de Janeiro, Brazil, approved the study protocol, participant information sheet, and consent inform. We have recruited thirty-six patients were recruited, 10 of which did not complete follow up. We excluded one of them from the research due to severe episode of hypoglycemia and subsequent interruption of insulin therapy. We evaluated all the participants at monthly intervals in the 6 months of the study and the assistant physician adjusted the dosage schedule of insulin therapy when necessary.

Short-Form 36 Health Survey (SF-36), Problem Areas in Diabetes (PAID) and Beck Depression Inventory (BDI) questionnaires, body mass index (BMI), waist circumference and glycosylated hemoglobin (HbA1c) have been recorded at baseline and 6 months after the beginning of insulin therapy. Socio-demographics characteristics (gender, marital status and educational level), presence of complications related to T2D and comorbidities were also registered. The ophthalmologist examined all patients and evaluated the presence/degree of diabetic retinopathy by fundoscopy in mydriasis. Neuropathy was clinically diagnosed. Sensitivity was tested by the Semmes-Weinstein pressure asthesiometer. The 24-hour urinary excretion of albumin (UAER) was measured. UAER was considered abnormal when it was > 30 mg/24 h. Microalbuminuria was defined as 30–300 mg/24 h and macroalbuminuria as > 300 mg/24 h. All patients with micro or macroalbuminuria were defined as having nephropathy. Hypoglycemia was defined as blood glucose < 50 mg/dL in the presence or not of clinical symptoms.

HRQoL was measured using a generic (Brazilian version of the SF-36 Health Survey: SF-36) and a disease-specific questionnaire (Problem Areas in Diabetes: PAID). SF-36 provides scores related to both the physical and mental dimensions of HRQoL. This scale consists of 36 items, which constitutes 8 domains: physical function (PF), role physical (RP), bodily pain (BP), general health perceptions (GH), vitality (VT), social functioning (SF), role emotional (RE), and mental health (MH). Scores ranged from 0 to 100, with higher scores indicating better quality of life in those dimensions [11]. The Brazilian edition of PAID consists of a 20-item self-report measure of diabetes-related emotional distress with high internal reliability, sensitivity to change, and clinical utility. It includes 20 questions that assess diabetes-related stressors. The score is rescaled from 0 to 100, with a high score indicating lower disease-specific HRQoL or greater emotional distress related to diabetes [12–14]. Portuguese version of the Beck Depression Inventory (BDI) reliably enables physicians of the presence and severity of depressive symptoms. It is based on the Diagnostic and Statistical Manual, 4th edition (DSM IV, 1994) criteria. The assessment has a minimum score of 0 and a maximum score of 63, with a high score indicating worse depressive symptoms. [15] Patients were excluded from the study if they had a history of chronic kidney disease stages III/IV, dementia, coronary or peripheral artery disease, organ transplant, cancer or use of anti-depressant medications.

### Statistical analysis

Data was analyzed using SAS 6.11 (SPSS Inc, Chicago, IL, USA). Numerical variables were expressed as mean, standard deviation (SD), median, minimum and maximum. Categorical data was expressed in percentage (%). The distribution of the sample was analyzed by Kolmogorov-Smirnoff test. Comparison between the two time periods (before/after insulin) has been performed by Wilcoxon test for the numerical variables and by McNemar’s test for categorical data. Non-parametric tests were used because the sample did not present a Gaussian distribution. To verify the existence of a significant association between range of scores (SF-36, PAID and BDI) and baseline categorical variables the Mann-Whitney’s test was used, and to verify the association with baseline numerical variables the Spearman’ s rank correlation coefficient (rs) was used. A p-value smaller than 0.05 on the two-tail was considered to indicate statistical significance.

## Results

Thirty-six T2D patients (67.9 % females, age 66.5 ± 5.1 years) were recruited, 26 of whom completed the follow-up. Generic (Short-Form 36 Health Survey - SF-36) and specific (Problem Areas in Diabetes - PAID) HRQoL questionnaires, Beck Depression Inventory (BDI), clinical, laboratorial and socio-demographic data were recorded at baseline and after 6 months of insulin therapy. Baseline characteristics of the sample are shown in Table 1.

**Table 1:** Baseline numerical and categorical variable of the sample (*n* = 26)

Numerical baseline	Median	Minimum	Maximum
Age (years)	66	60	79
BMI (Kg/m^2^)	28.1	21	41.3
Waist circumference (cm)	100.5	76	129
Hb A1C (%)	8.7	7.6	13.2
Number of medications	7	3	14
Years of T2D	8.5	1	37
Categorical baseline	N	%	
Gender (M/F)	7/19	36/64	
Marital status			
Single	4	15.3	
Married	16	61.5	
Widowed	6	23.2	
Educational level			
Iliterate	8	28.6	
Primary	8	28.6	
Secondary ou further	10	42.8	
Retinopathy	12	46.1	
Neuropathy	17	65.3	
Nephropathy	13	50	
Hypertension	26	100	
Hypoglycemia	8	30.7	
Insulin scheme			
Bedtime (NPH)	15	57.6	
Basal	10	38.4	
Basal-bolus	1	4.0	
Insulin prescribed			
NPH	25	96.1	
Detemir	1	3.84	
Aspart	1	3.84	

*SD* standard deviation

The mean in BDI score was 10.5, similar to other report in a Brazilian sample, in which the mean was 10.0 (*p* = 0.07). So we could infer that our sample was relatively representative of our population [15]. There was a significant reduction in HbA1c levels (8.7 vs 7.9; *p* = 0.019). There were no significant changes in BMI (28.1vs 28.3; *p* = 0.62) and waist circumference (100.5 vs 99.5; p =0.77). There was a significant reduction in BDI score when compared to baseline values (10.5 vs 7.0; *p* = 0.008), indicating an improvement in depressive symptoms. Despite the robust drop in BDI score, HRQoL did not change significantly in any of domains when the total sample was analyzed as a whole, as shown in Table 2.

**Table 2:** Anthropometric variables, BDI, SF-36 and PAID scores before and after insulin therapy

Variable	Before median	After median	*p*	Range (mean)
BMI (Kg/m^2^)	28.1	28.3	0.62	0.13
Waist circumference (cm)	100.5	99.5	0.77	−0.36
Hb A1C (%)	8.7	7.9	0.019	−0.84
BDI	10.5	7.0	0.008	−3.61
Physical functioning	52.5	55.0	0.68	1.25
Role-physical	25.0	50.0	0.13	8.93
Bodily pain	51.5	61.0	0.31	5.79
General health	53,5	52.0	0,34	4.96
Vitality	52.5	57.5	0.47	2.14
Social functioning	75.0	87.5	0.43	4.91
Role- emotional	66.6	83.3	0.74	3.57
Mental health	68	70.0	0.45	1.71
Mental health	68	70.0	0.45	1.71
PAID	20.5	16.5	0.23	−4.11

*BMI* body mass index, *HbA1C* hemoglobin glycated, *BDI* Beck depression inventory, *PAID* Problem areas in diabetes

However, when correlation between the range of scores (BDI, SF-36 and PAID), baseline variables and their respective ranges has been assessed, it was shown that there was an negative correlation between BDI range and physical functioning (*rs* = −0.393; *p* = 0.038), physical role (*rs* = −0.394; *p* = 0.038) and pain ranges (*rs* = −0.427; *p* = 0.023) as well between baseline BDI score and BDI range (*rs* = −0.608; *p* = 0.001). Otherwise, there was a positive correlation between the BDI range and PAID range (*rs* = 0.397; *p* = 0.037).

We also observed a trend of positive correlation between the range of BDI score and HbA1c levels (*rs* = 0.337; *p* = 0.080, which means that the higher the reduction in HbA1c levels, the higher the improvement in depressive symptoms.

When gender and educational level have been taken into account, It was observed that men had a greater improvement in physical functioning domain than women (12.8 + 6.0 vs −4.2 + 3.7; *p* = 0.018) as well those with higher educational level did in mental health domain when compared with those with lower levels (7.4 + 5.2 vs −3.2 + 2.6; *p* = 0.017).

As far as chronic complications related to T2D are concerned, participants with or without retinopathy or neuropathy did not present any statistical difference in the BDI (−2.9 + 2.0 vs - 4.2 + 1.6; *p* = 0.55) and HRQoL results (PAID: −0.5 + 5.8 vs −7.2 + 3.0; *p* = 0.41). However, participants with nephropathy had a lower range in physical functioning than those without nephropathy (7.7 + 12.1 vs 10.0 + 6.8; *p* = 0.010). Data is shown in Table 3.

**Table 3:** Correlation between BDI, SF-36 and PAID range and baseline numerical variables and their respective ranges

	Variable		Absolute range (after-before)								
			BDI	Physical functioning	Role physical	Pain	General health	Vitality	Social functioning	Role emotional	Mental health
Baseline	Age	r_s_	0.139	−0.084	0.041	−0.323	0.285	0.026	−0.284	−0.305	0.052
	BMI	r_s_	−0.201	0.158	0.301	−0.058	0.161	0.149	−0.061	0.046	0.235
	Waist circumference	r_s_	−0.167	0.204	0.203	−0.182	0.105	0.278	−0.028	−0.166	0.179
	Hb A1C	r_s_	−0.121	−0.092	0.082	0.093	0.287	−0.018	−0.050	0.138	0.238
	BDI	r_s_	−0.608	−0.039	0.268	0.250	0.107	−0.048	0.076	0.012	−0.118
		p	0.001								
Range (after/before)	BMI	r_s_	0.276	−0.098	−0.142	−0.103	0.090	0.014	−0.022	0.074	0.010
	Waist circumference	r_s_	0.196	0.008	−0.141	−0.363	0.003	0.162	−0.047	0.214	0.134
	HbA1C	r_s_	0.337	−0.121	−0.221	−0.149	−0.306	−0.051	0.050	−0.033	−0.132
	BDI	r_s_		−0.393	−0.394	−0.427	−0.347	−0.309	−0.368	−0.162	−0.288
		p		0.038	0.038	0.023	0.071	0.11	0.054	0.41	0.14

*r*_*s*_: Spearman’ s rank correlation coefficient; *p*: value. p values not shown did not achieved statistical significance

## Discussion

This is to the best of our knowledge the first research that evaluates prospectively the association of starting insulin therapy with HRQoL and depressive symptoms specifically in elderly with T2D. Given the unique characteristics of this population (elevated prevalence of cardiovascular disease, frailty, increased risk of falls, more deleterious consequences from a hypoglycemic episode), it is of value that studies evaluating this specific population are conducted. Indeed, this population is more vulnerable to lose their independence and the introduction of insulin therapy increases treatment complexity, requiring some cognitive and visual skills [2].

We observed that starting insulin therapy in elderly participants with T2D was associated with significant improvement in depressive symptoms, as indicated by an important reduction in BDI score. We also found that the higher the improvement in depressive symptoms, the higher the improvement in physical functioning and role physical, as well as in pain domain. The influence of depressive symptoms in HRQoL of people with T2D is known and has been published elsewhere [16]. Recent data indicated a higher prevalence of depression in a Brazilian population with T2D and a consequent reduction in its HRQoL, most notably in physical functioning and physical role. However, this study evaluated subjects aged between 40–60 years. Therefore, we can not extrapolate these data [17]. Another study examined the relationship between severity of depressive symptoms and diabetes-related emotional distress in patients with T2D and evaluated whether this relationship is independent of demographic and clinical characteristics, such as sex, age, duration of diabetes, glycemic control, and diabetic complications. This study used the same questionnaires used in our research (SF-36, PAID and BDI). The results indicated that diabetes-related emotional distress (PAID) was significantly related to the severity of depressive symptoms. On multivariate analysis by regression model, it was found that this relation was independent of the demographic, including age, clinical characteristics, and therapeutic regimens [18].

We found a direct correlation between the BDI and PAID ranges, which signifies the higher the improvement on depressive symptoms, the bigger the improvement on HRQoL complaints registered in PAID score. We also found an inverse correlation between baseline BDI score and BDI range, which means the more severely depressed the individual at baseline, the smaller the improvement on depressive symptoms.

We did not find a clear change in HRQoL scores. However, when gender and educational level have been taken into account, we also observed that men had a greater improvement in physical functioning domain than women. This gender interaction with HRQoL has also been showed previously by Eljedi et al. His group have studied a sample of patients with T2D and evaluated HRQoL in this population in a cross-sectional design. Their results indicated that HRQoL of patients with T2D was strongly reduced and indeed identified that women were specially affected [19]. In Zhang’s study, it has also been identified an interaction between HRQoL and gender, and women were more severely affected [20]. Likewise, women did worse than men in Wandel’s review and in Akinci’s study [21, 22].

Educational level seems also to have a different meaning on our population. Participants with higher educational levels had a greater improvement in mental health when compared with those with lower educational levels, as previously shown [23]. Our patients with nephropathy had a worse outcome in physical functioning when compared to those without this comorbidity. Although these data have to be reassured, there is some available evidence indicating that proteinuria per se plays a role in HRQoL of patients with diabetic nephropathy [24].

Finally, we observed a trend of direct correlation between the range of BDI score and HbA1c levels, which means that the higher the reduction in HbA1c levels, the higher the improvement in depressive symptoms. However, this did not achieve statistical significance, possibly because of relatively small sample.

Indeed our data showed reduction in HbA1c levels, although of smaller magnitude than in non-elderly adult population studies, presumably because of the lower doses used in our sample (related to less strict glycemic targets) and the relatively short follow-up [4–7]. We did not observe an increase in body weight as described in other studies possibly for the same reasons [25].

The major limitation of our study is its relatively small sample size that could have influenced the statistical power to show a significant change in HRQoL scores. However, the number of individuals included is similar to the studies published previously [16, 26]. We adopted a convenience sample and have taken into account the study of Kamoi and cols, in which a prospective comparison of different types of insulin scheme has been performed, as a model. They have studied a smaller number of subjects than our study and have showed that HRQoL of patients treated by continuous subcutaneous insulin therapy is superior to that treated by multiple daily insulin injections (*p* < 0.05). The results in SF-36 and PAID scores were very similar, when whole sample was analyzed, which makes improbable that if the sample was greater we would find significant differences. However, it is important to note that the exclusion of the case of serious hypoglycemia may have influenced the results. The participants lost in the follow up maybe could have been those in whom insulin therapy had a different meaning. It could be also considered a bias. Furthermore, the design of the study (lack of a control-group and the open-label format) does not guarantee that the increase in BDI score was related to insulin therapy or to the improvement in glycemic control. A possible improvement in depressive symptoms related to the starting of insulin therapy in elderly diabetic subjects could be relevant in clinical setting in which starting insulin faces some barriers, especially in elderly subjects. However, these data must be investigated in a randomized controlled study and further research is warranted.

## Conclusions

In our study, the beginning of insulin therapy in elderly participants with T2D was associated with a significant improvement in depressive symptoms and did not seem to affect adversely the HRQoL of this population, both fundamental aspects when deciding for any therapy directed to people with T2D.
